# Predictive Validity and Classification Accuracy of ActiGraph Energy Expenditure Equations and Cut-Points in Young Children

**DOI:** 10.1371/journal.pone.0079124

**Published:** 2013-11-11

**Authors:** Xanne Janssen, Dylan P. Cliff, John J. Reilly, Trina Hinkley, Rachel A. Jones, Marijka Batterham, Ulf Ekelund, Søren Brage, Anthony D. Okely

**Affiliations:** 1 Interdisciplinary Educational Research Institute, University of Wollongong, Wollongong, New South Wales, Australia; 2 School of Psychological Sciences and Health, University of Strathclyde, Glasgow, Scotland; 3 Centre for Physical Activity and Nutrition Research (C-PAN), Deakin University, Melbourne, Victoria, Australia; 4 Centre for Statistical and Survey Methodology, University of Wollongong, Wollongong, New South Wales, Australia; 5 Department of Sport Medicine, Norwegian School of Sport Sciences, Ullevål Stadion, Oslo, Norway; 6 MRC Epidemiology Unit, Institute of Metabolic Science, Addenbrooke’s Hospital, Cambridge, United Kingdom; University of Sao Paulo, Brazil

## Abstract

**Objectives:**

Evaluate the predictive validity of ActiGraph energy expenditure equations and the classification accuracy of physical activity intensity cut-points in preschoolers.

**Methods:**

Forty children aged 4–6 years (5.3±1.0 years) completed a ∼150-min room calorimeter protocol involving age-appropriate sedentary, light and moderate-to vigorous-intensity physical activities. Children wore an ActiGraph GT3X on the right mid-axillary line of the hip. Energy expenditure measured by room calorimetry and physical activity intensity classified using direct observation were the criterion methods. Energy expenditure was predicted using Pate and Puyau equations. Physical activity intensity was classified using Evenson, Sirard, Van Cauwenberghe, Pate, Puyau, and Reilly, ActiGraph cut-points.

**Results:**

The Pate equation significantly overestimated VO_2_ during sedentary behaviors, light physical activities and total VO_2_ (P<0.001). No difference was found between measured and predicted VO_2_ during moderate-to vigorous-intensity physical activities (P = 0.072). The Puyau equation significantly underestimated activity energy expenditure during moderate-to vigorous-intensity physical activities, light-intensity physical activities and total activity energy expenditure (P<0.0125). However, no overestimation of activity energy expenditure during sedentary behavior was found. The Evenson cut-point demonstrated significantly higher accuracy for classifying sedentary behaviors and light-intensity physical activities than others. Classification accuracy for moderate-to vigorous-intensity physical activities was significantly higher for Pate than others.

**Conclusion:**

Available ActiGraph equations do not provide accurate estimates of energy expenditure across physical activity intensities in preschoolers. Cut-points of ≤25counts⋅15 s^−1^ and ≥420 counts⋅15 s^−1^ for classifying sedentary behaviors and moderate-to vigorous-intensity physical activities, respectively, are recommended.

## Introduction

Measuring young children’s physical activity (PA) and sedentary behavior (SB) objectively is important to improve all aspects of PA-related research in this age group. Accelerometry has become the method of choice to objectively assess children’s free-living habitual PA and SB and the ActiGraph accelerometer is the most widely used in young children [1]–[3]. Although accelerometry is becoming more widely used among young children, this method is not without limitations. Several equations [4], [5] and cut-points [4]–[9] have been developed to predict energy expenditure (EE) and classify PA intensity or SB from ActiGraph accelerometer output counts per time unit. The accuracy of these equations for predicting EE over the range of PA intensities is, however, unclear. Differences in EE equations [4], [5] and PA intensity cut-points [4]–[9] exist. Differences may be due to the methods used to develop these equations and/or cut-points [4]–[9]. Some studies have used EE measured by indirect calorimetry as the criterion measure [4]–[6], whereas others have used direct observation [7]–[9] sometimes using different instruments or criteria to define PA intensity. In addition, there are differences in the age ranges examined, and activities included in the validation protocols vary from using only ambulatory activities (walking and running) [4] to including free-living activities (e.g. arts and crafts and stair walking) [5]–[9].

Applying different cut-points results in substantial differences in the estimated time children spend in different intensities of PA. These inconsistencies make it difficult to compare findings between studies [9]–[11] and to determine the extent to which young children are physically active and meet PA guidelines [1]. To establish which, if any, equations and cut-points are most accurate, they need to be simultaneously cross-validated in an independent sample of children using a standardized activity protocol and appropriate criterion measures. To our knowledge, there are no studies demonstrating the most accurate equations and cut-points among preschool children. Therefore, the aims of this study were to: 1) examine the predictive validity of ActiGraph EE equations; and 2) compare the classification accuracy of ActiGraph cut-points for classifying SB and PA intensity, in 4–6 year-olds.

## Methods

### Ethics Statement

The study was approved by the University of Wollongong/South Eastern Sydney and Illawarra Area Health Service Human Research Ethics Committee. Parents provided informed written consent, and their children provided their verbal assent to participate in the study.

### Study Participants

Participants were recruited from the Illawarra region of New South Wales, Australia. Children were excluded from the study if they had a disease known to influence their energy balance, had a physical disability and/or were claustrophobic.

### Protocol

During a first visit to the university participants were familiarized with the room calorimeter and the activity protocol. A second visit occurred within a week after the first visit. Parents were asked to give their child a standardized breakfast 1.5 h before entering the room calorimeter as it was considered unfeasible to ask young children to fast overnight before completing a 2.5-h activity protocol in a room calorimeter. Participants followed a 150-min activity protocol within the room calorimeter. This included child-appropriate activities involving SB, light-intensity physical activity (LPA) and moderate-to vigorous-intensity physical activity (MVPA). All children were guided through the protocol by a research assistant and performed all activities in an identical order over a pre-determined duration as described in Table 1. Children were encouraged to move immediately from one activity to the other. However, if children required a rest, they were allowed to have a break. Start and end times of these breaks were noted down and removed from the data for analysis.

**Table 1:** Room calorimetry protocol.

Activity	Time (min)
*Sedentary Intensity*	
Watching TV–sitting in a beanbag	30
Talking on telephone with parents – sitting	2
Reading books with a cassette – sitting	5
Drawing/colouring in – sitting	10
**Subtotal**	**47**
*Light Intensity*	
Playing with toys, Lego, dolls, puzzles, games – sitting on floor	20
Drawing on a whiteboard – standing	3
Personal grooming (brushing teeth, hair, washing hands/face)	3
Dressing up in costumes	5
Playing musical instruments – standing	5
Domestic chores (hanging out washing, setting table)	4
Mini-golf	5
Walking on spot – light effort (Wii game)	2
Playing quoits	3
**Subtotal**	**50**
*Moderate and vigorous intensity*	
Cleaning (packing away toys, dusting, sweeping)	5
Running on spot – moderate effort (Wii game)	5
Hopscotch, star jumps, walking stairs	5
Shooting small basketball into small ring on wall	3
Animal walks (e.g., like a chicken, kangaroo, bear)	5
Wii sports cycling	10
Hitting a balloon in the air and catching it	5
Circuit (walking up foam stairs, jumping off, crawling through a standing hoop, and running back)	5
Running on the spot (Wii game)	5
Dancing/aerobics (Wii Game)	2
**Subtotal**	**50**
**GRAND TOTAL**	**147**

### Room Calorimeter

Oxygen consumption (VO_2_) and carbon dioxide production (VCO_2_) were measured continuously (paramagnetic O_2_ and infrared CO_2_ analyzers, Sable System Inc, Las Vegas USA) and corrected to standard temperature, pressure and humidity in the room calorimeter (3 m×2.1 m×2.1 m) at the University of Wollongong. Technical procedures are described in more detail elsewhere [12]. Chamber air was sampled every two minutes and rates of O_2_ consumption and CO_2_ production were then averaged over 10-min blocks to produce stable measures of EE [13]. EE for every 10-min block was calculated using the Weir equation [14]. Individualized multiples of resting EE (METs) were calculated by dividing measured EE for each child by their individually estimated basal metabolic rate (BMR) using the Schofield equation for children aged 4–10 years [15]. The 10-min blocks of EE were classified based on their equivalent MET values, into PA intensities as follows; SB ≤1.5 times predicted BMR, LPA 1.5 to 3.0 times predicted BMR and MVPA ≥3.0 times predicted BMR. Activity energy expenditure (AEE) was calculated by deducting BMR from measured EE.

### Direct Observation of PA Intensity

Each child was videotaped during their time in the room calorimeter and activity start and end times, breaks and transitions were recorded. PA intensity was classified based on the Children’s Activity Rating Scale (CARS) [16]. CARS is based on a 1 to 5 coding scheme and is a reliable and valid tool to assess PA levels in young children [16]. It has been used in several accelerometer validation studies in young children [9], [17]. Video footage was coded using Vitessa 0.1 (Version 0.1, University of Leuven, Belgium). Data were coded by one observer who undertook two days of CARS training. After coding, a weighted average CARS score was calculated by multiplying each numeric activity code by the percentage of 15 s or 60 s in that time interval and summing the products. Averaged epochs were classified into intensity categories using the CARS criteria: SB <level 2.0; LPA ≥level 2.0 and ≤3.0; MVPA >level 3.0 [18].

### Accelerometry

The ActiGraph GT3X (ActiGraph Corporation; Pensacola USA) uses a solid-state triaxial accelerometer. In this study only the vertical axis was used as the cut-points and equations included for testing were developed based on accelerometer counts from the vertical axis. Before each experiment the accelerometer was initialized to collect data in 15-s epochs. Before entering the room calorimeter children were fitted with an ActiGraph GT3X which was worn on the right mid-axillary line of the hip and secured with an elastic belt.

### Data Reduction

#### Prediction of EE

ActiGraph counts were converted to AEE or VO_2,_ using the Puyau (PU) or Pate (PT) equations according to the specified units in the equation [4], [5] (Table 2) and averaged over 10-min blocks. To adjust for the high *y*-intercepts of the equations, a flex-point of 25 counts per 15 s, which is a commonly used SB cut-point, was used [6]. This meant that whenever counts per 15 s were <25 predicted EE values were assigned AEE = 0 kJ⋅kg^−1^⋅min^−1^, or VO_2_ = 9.1 ml⋅kg^−1^⋅min^−1^ depending on the equation used [4], [5]. Participants’ predicted and measured EE data were averaged per intensity and over the duration of the protocol. Predicted EE values were then compared to measured EE values by the room calorimeter.

**Table 2:** ActiGraph cut-points and equations for children.

Author	Sample	Criterion measure	Activities		Equation/Cut-point						
					counts⋅15 s^−1^				counts⋅60 s^−1^		
Evenson et al. [6]	n = 33	Portable metabolic system	Sit, watch TV, colouring in,	SB	≤25				≤100		
	Age = 5–8 years		slow walk, stair climbing	LPA	>25				>100		
	Mean age = 7.3 years		dribble basketball, brisk	MVPA	≥574				≥2296		
	21 girls, 12 boys		walk, bicycling, jumping,								
			jacks, running.								
Sirard et al. [8]	n = 33	Direct observation (CARS)	Sitting, sitting and playing,		Age 4:		Age 5:		Age 4:	Age 5:	
	Age = 5–8 years		slow walking, fast walking,	SB	≤363		>398		≤1452	≤1592	
	Mean age = 7.3 years		jogging.	LPA	>363		≤398		>1452	>1592	
	21 girls, 12 boys			MVPA	≥813		≥891		≥3252	≥3564	
v. Cauwenberghe et al. [9]	n = 18	Direct observation (CARS)	Sitting, standing, drawing,	SB	≤372				≤1488		
	Age = 4–6 years		walking, jogging at seven	LPA	>372				>1488		
	Mean age = 5.8 years		speed levels, free play	MVPA	≥585				≥2340		
	10 girls, 8 boys		session								
Pate et al. [4]	n = 29	Portable metabolic system	Rest, slow walking, brisk	VO_2_ = 10.0714+0.02366 × counts⋅15 s^−1^							
	Age = 3–5 years		walk and running.	SB	≤37			≤148			
	Mean age = 4.4 years			LPA	>37			>148			
	16 girls, 13 boys			MVPA	≥420			≥1689			
Puyau et al.^‡^ [5]	N = 26	Whole room calorimetry	Computer games, arts and	AEE = 0.0183+0.000010 × counts⋅60 s^−1^							
	Age = 6–16 years		crafts, playing with toys,	SB	≤199			≤799			
	Mean age = 10.7 years		walking, martial arts,	LPA	>199			>799			
	12 girls, 14 boys		running, jumping a rope,	MVPA	≥799			≥3199			
			skipping, soccer.								
Reilly et al.^‡^ [7]	N = 30	Direct observation (CPAF)	No structured activities.	SB	≤274			≤1099			
	Age = 3–4 years			LPA	NA			NA			
	Mean age = 3.7 years			MVPA	NA			NA			
	10 girls, 20 boys										

SB, Sedentary behaviour; LPA, Light physical activity; MVPA, Moderate-to-vigorous physical activity ^‡^ developed as counts⋅60 s^−1^ all others were developed as counts⋅15 s^−1.^

#### Prediction of physical activity intensity

ActiGraph output and direct observation data were used as 15-s epochs or converted to 60-s epochs depending on the cut-point used. ActiGraph data were classified as SB, LPA, or MVPA using ActiGraph cut-points defined by Evenson (EV), Sirard (SI), van Cauwenberghe (CB), Reilly (RE), PT and PU [4]–[9] (Table 2) and aligned with the criterion epochs. Epochs were excluded from data analyses if they were part of a break between activities or the child was off screen in the direct observation videos. Reilly et al. only examined SB and therefore no LPA or MVPA cut-point was available [7]. EV and PU were developed in older children, however, EV has been shown to be most accurate in 5–15 year-olds and was therefore included [6]. The PU cut-point has been used extensively in preschool studies [19]–[21].

**Figure 1:**
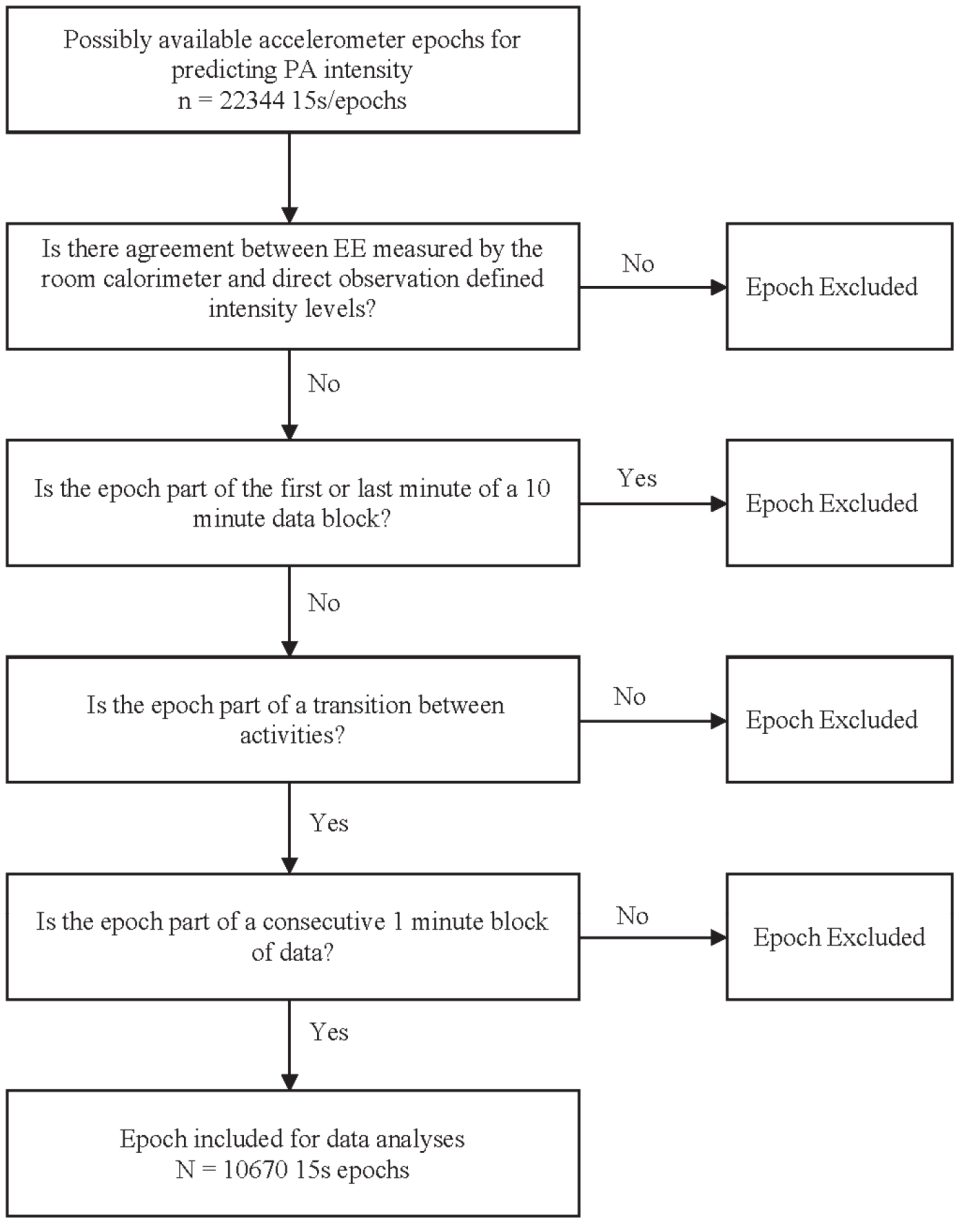
Selection procedures for including valid epochs to determine the classification accuracy of ActiGraph cut-points for defining physical activity intensity.

The required EE for a given activity varies between individual children [4], [22]. Because direct observation systems such as CARS rely on subjective classification and use general category descriptions to assign levels to activities based on the apparent intensity of the activity, it is possible that misclassification may occur for some individuals. To overcome this potential limitation and confirm findings for PA intensity classification based on direct observation, we developed an additional criterion measure including both direct observation and EE measured by the room calorimeter. Ten-minute average EE values were divided by predicted BMR to define intensity levels. Each of the forty 15-s epochs within the 10 min immediately prior to the measured average EE value were classified as SB, LPA, or MVPA. Direct observation data and EE data were compared for every 15-s or 60-s epoch. Thereafter, criterion epochs were excluded if PA intensity defined using EE measured by the room calorimeter did not agree with the intensity levels derived via direct observation. That is, agreement was established if both measures provided the same intensity classification (e.g. for SB measured EE and the weighted CARS value had to be ≤1.5 METs and<level 2, respectively).In addition, to ensure that any small time lag in the calorimeter readings would not lead to mismatching criterion data with accelerometer data, epochs within the first and last minute of a 10-min EE data block were excluded. Likewise, criterion epochs which were part of a break between activities were excluded. Last, criterion epochs were excluded if they were not part of at least four consecutive 15-s epochs within which children were active at a consistent intensity (Figure 1). ActiGraph data were classified as described using procedures consistent with the direct observation only analysis. Classified ActiGraph data were then compared with criterion epochs derived from combining measured EE and direct observation data.

### Statistical Analysis

Measured EE and predicted EE were compared using dependent *t*-tests with a Bonferroni adjustment for multiple comparisons (i.e. SB, LPA, MVPA and AEE; P<0.0125). In addition, to assess the variance in the difference between predicted versus measured energy expenditure within subjects the coefficient of variation (CV) was calculated for each activity level. This was done by dividing the mean difference by the standard deviation per participant for each intensity. To evaluate classification accuracy, sensitivity (Se), specificity (Sp), and area under the receiver operating curve (ROC-AUC) were calculated. ROC-AUC values were defined as excellent (0.9–1.0), good (0.8–0.9), fair (0.7–0.8), or poor (<0.7) [23]. All statistical analyses were performed using STATA Version 12 (StataCorp, College Station, USA).

## Results

Of the 44 children enrolled in the study, four ended their participation early due to illness (n = 1); inability to schedule a second visit (n = 1); or refusal to participate in the activity protocol (n = 2). Of the 40 children who completed both visits, two had missing data due to calorimeter malfunction. For the remaining 38 children, 33 (86.8%), 36 (94.7%), and 34 (89.5%) had at least one 10-min block of SB, LPA, and MVPA, respectively, according to measured EE values. Descriptive characteristics are presented in Table 3.

**Table 3:** Participant characteristics.

	Total sample (n = 40)	Boys (n = 22)	Girls (n = 18)
Age (years)	5.3±1.0	5.2±1.0	5.3±1.1
Height (cm)	112.7±8.1	114.3±6.2	110.9±9.7
Weight (kg)	20.6±3.7	21.5±2.4	19.4±4.6
BMI (kg/m^2^)	16.1±1.5	16.5±1.3	15.5±1.6
Predicted BMR (kcal/kg/min)	0.032±0.003	0.032±0.002	0.032±0.004
% overweight*	25.0	27.2	22.2

Values are mean ± SD; *defined according to Cole et al. [34].

### Prediction of EE

**Figure 2:**
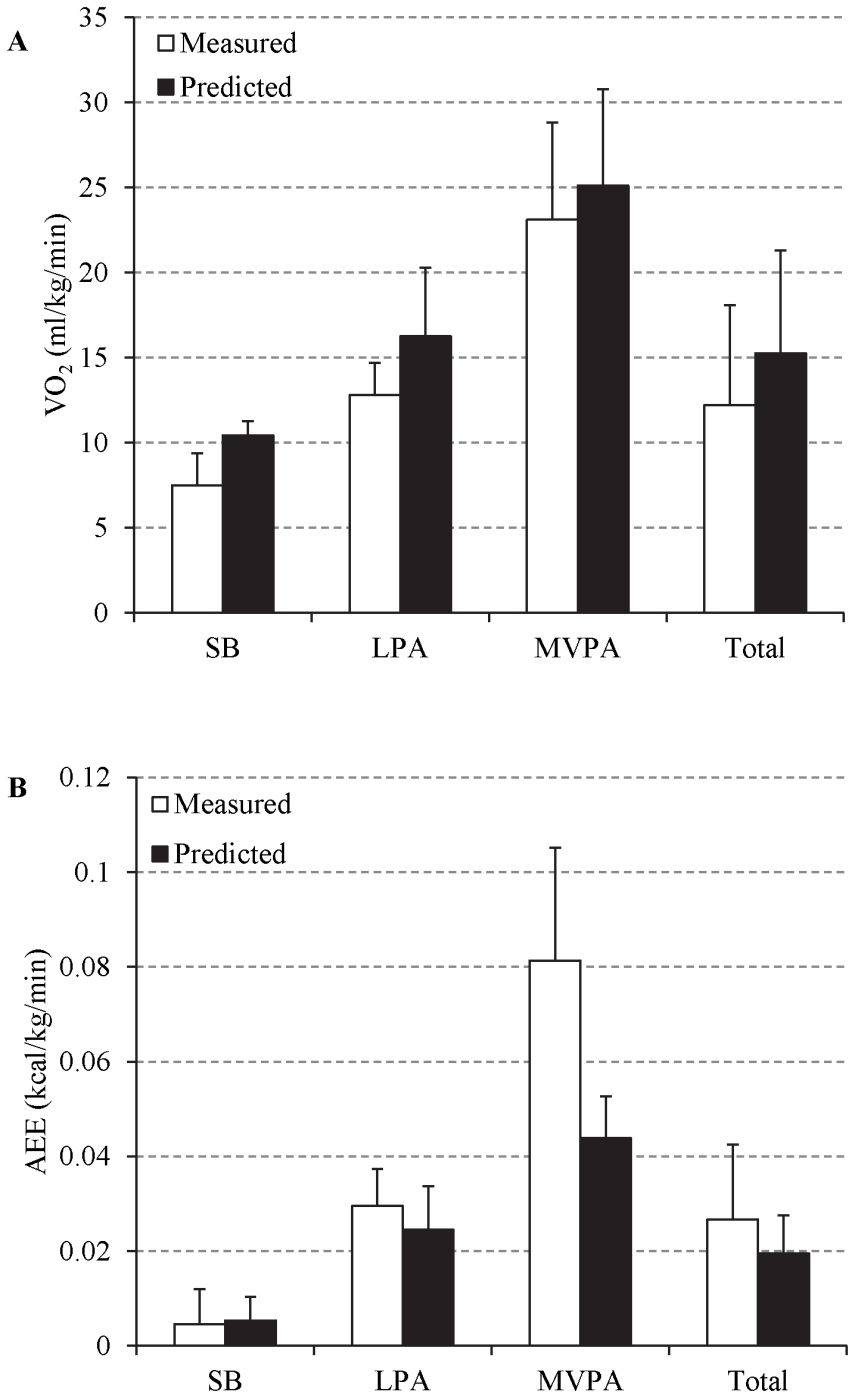
Measured versus predicted mean energy expenditure values (±SD) for the Pate (A) and Puyau (B) equations. *Statistically significant (P<0.0125).

Observed and predicted VO_2_ and AEE values for the PT and PU equations are shown in Figures 2A and B. The PT equation significantly overestimated VO_2_ during SB and LPA and for total VO_2_ (P<0.001) but did not show a significant difference between measured and predicted VO_2_ during MVPA (P = 0.072). However, at individual level the CV was 52.9%, 78.0%, 67.5%, and 91.3% for SB, LPA, MVPA, and total VO_2_ respectively. The PU equation significantly underestimated AEE during MVPA and LPA and for total AEE (P<0.0125) but did not show a significant difference for activity energy expenditure during SB (P = 0.5481). For SB, LPA, MVPA, and total AEE the CV was 70.5%, 75.5%, 44.1%, and 98.8% respectively.

### Prediction of PA Intensity

Table 4 reports the total numbers of epochs included when using direct observation alone and combined direct observation and measured EE as the criterion measure. Using direct observation alone as the criterion measure, classification accuracy for SB was good and significantly higher for EV compared to all others (P<0.05). For LPA, all cut-points exhibited poor classification accuracy. However, classification accuracy was significantly higher for EV compared to all others (P<0.05). For MVPA, using the PT cut-point resulted in fair classification accuracy which was significantly higher compared to all others (P<0.05). Results are reported in Table 5.

**Table 4:** Included data.

	Included epochs when using direct observation as criterion measure% (number of epochs included)	Included epochs when using direct observation combined with EE as criterion measure.% (number of epochs included)
SB	96.3 (6881)	57.5 (4108)
LPA	96.4 (7325)	65.1 (4945)
MVPA	62.5 (4747)	21.3 (1617)
Total	84.8 (18953)	47.8 (10670)

**Table 5:** Sensitivity (Se%), Specificity (Sp%) and area under the ROC curve (ROC-AUC) for the classification of SB, LPA and MVPA using direct observation as criterion measure.

		SB			LPA			MVPA	
	Se% (95% CI)	Sp% (95% CI)	ROC-AUC (95% CI)	Se% (95% CI)	Sp% (95% CI)	ROC-AUC (95% CI)	Se% (95% CI)	Sp% (95% CI)	ROC-AUC (95% CI)
v. Cauwenberghe [9]	98.2	31.5	0.65	9.2	93.9	0.52	45.0	92.8	0.69
	(97.8–98.5)	(30.7–32.2)	(0.64–0.65)	(8.6–9.7)	(93.5–94.3)	(0.51–0.52)	(43.6–46.4)	(92.5–93.2)	(0.68–0.70)
Evenson [6]	86.7	72.9	0.80	54.8	74.8	0.65	45.7	92.7	0.69
	(85.8–87.5)	(72.1–73.6)	(0.79–0.80)	(53.8–55.7)	(74.0–75.6)	(0.64–0.65)	(44.3–47.1)	(92.3–93.0)	(0.69–0.70)
Pate [4]	89.2	67.3	0.78	42.8	81.7	0.62	54.2	88.9	0.72
	(88.4–89.9)	(66.6–68.0)	(0.78–0.79)	(41.8–43.7)	(81.0–82.4)	(0.62–0.63)	(52.8–55.7)	(88.4–89.3)	(0.71–0.72)
Puyau [5]	97.3	47.2	0.72	30.9	81.3	0.56	31.5	96.8	0.64
	(96.4–98.0)	(45.6–48.8)	(0.71–0.73)	(29.2–32.7)	(79.8–82.7)	(0.55–0.57)	(28.9–34.2)	(96.2–97.3)	(0.63–0.65)
Sirard [8]	98.3	29.9	0.64	13.6	87.1	0.50	27.1	96.7	0.62
	(98.0–98.6)	(29.2–30.6)	(0.64–0.65)	(13.0–14.2)	(86.5–87.7)	(0.50–0.51)	(25.8–28.4)	(96.4–96.9)	(0.61–0.63)
Reilly [7]	98.2	39.2	0.69						
	(97.4–98.7)	(37.7–40.8)	(0.67–0.70)						

When combining direct observation with measured EE as criterion measure results were slightly inflated compared to using direct observation alone. Classification accuracy for the EV cut-point was excellent for SB and fair for LPA and MVPA. The EV cut-point showed significantly higher accuracy compared to all others except the PT cut-point. PT showed the highest classification accuracy for MVPA. Results for each cut-point using the combined criterion measure are reported in Table 6.

**Table 6:** Sensitivity (Se%), Specificity (Sp%) and area under the ROC curve (ROC-AUC) for the classification of SB, LPA and MVPA using EE combined with direct observation as the criterion measure.

			SB			LPA			MVPA	
	Se% (95% CI)	Sp% (95% CI)		ROC-AUC (95% CI)	Se% (95% CI)	Sp% (95% CI)	ROC-AUC (95% CI)	Se% (95% CI)	Sp% (95% CI)	ROC-AUC (95% CI)
v. Cauwenberghe [9]	99.9	39.9		0.70	13.1	96.2	0.55	59.7	91.3	0.75
	(99.7–100.0)	(38.7–41.1)		(0.69–0.71)	(12.2–14.1)	(95.6–96.6)	(0.54–0.56)	(57.2–62.1)	(90.7–91.8)	(0.75–0.76)
Evenson [6]	90.7	89.7		0.90	69.9	82.2	0.76	60.5	91.0	0.76
	(89.8–91.6)	(89.0–90.5)		(0.90–0.91)	(68.6–71.2)	(81.2–83.2)	(0.75–0.77)	(58.1–63.0)	(90.4–91.6)	(0.75–0.77)
Pate [4]	92.4	86.2		0.89	56.2	86.0	0.71	69.6	86.4	0.78
	(91.6–93.2)	(85.4–87.0)		(0.89–0.90)	(54.9–57.6)	(85.0–86.9)	(0.70–0.72)	(67.3–71.8)	(85.3–86.8)	(0.77–0.79)
Puyau [5]	98.6	60.7		0.80	44.3	87.2	0.66	44.9	95.8	0.70
	(97.6–99.2)	(58.3–63.1)		(0.78–0.81)	(41.5–47.2)	(85.4–88.9)	(0.64–0.68)	(40.0–50.0)	(94.9–96.6)	(0.69–0.72)
Sirard [8]	99.9	39.5		0.70	21.5	90.3	0.56	39.4	96.2	0.68
	(99.7–99.9)	(38.3–40.7)		(0.69–0.71)	(20.3–22.7)	(89.5–91.1)	(0.55–0.57)	(37.0–41.8)	(95.8–96.6)	(0.67–0.69)
Reilly [7]	99.1	50.3		0.75						
	(98.3–99.6)	(47.8–52.7)		(0.73–0.76)						

## Discussion

This study compared the validity of ActiGraph equations and cut-points for predicting EE and classifying PA intensity in young children. Although PT performed reasonable well predicting EE during MVPA, overall it significantly overestimated EE. Notably, neither equation - PT or PU - performed equally well across all intensities at either group or individual levels. These findings are consistent with a previous study, which reported that the PU equation underestimated individual total EE in 3–6 year-olds [24]. In addition, a study conducted in 5–15 year-olds reported significant differences in predicted versus measured EE during a variety of activities using the PU equation [22]. Considering the results of this and previous studies, we do not recommend the use of current ActiGraph equations for predicting EE over the whole range of physical activity intensities in young children. However, when interested in energy expenditure during MVPA, the PT equation could possibly be used. Nevertheless, further assessment in a broader range of typical non-ambulatory activities is required for the equations to be used with confidence across a broad range of free-living physical activity.

The EV cut-point showed significantly higher classification accuracy for SB, and the PT cut-point showed significantly higher classification accuracy for MVPA than all others. When using direct observation and measured EE simultaneously as criterion measure, EV did not differ significantly compared to PT. This is possibly due to the strict inclusion criteria when using the combined criterion measure which resulted in fewer epochs. For MVPA, the findings were consistent when using the combined direct observation and measured EE as criterion measure.

To our knowledge this is the first study to examine the classification accuracy of ActiGraph PA and SB cut-points in 4–6 year-olds. Trost et al. evaluated several cut-points in 5–15 year-olds and found that the cut-point of ≤25 counts⋅15 s^−1^ for SB resulted in excellent classification accuracy in that age range [22]. Results from the current study are similar and indicate that using the ≤25 counts⋅15 s^−1^ (EV) provided good classification accuracy of SB in 4–6 year old children. For MVPA classification accuracy was highest for the PT cut-point in 4–6 year old children. This finding is consistent with previous studies. In toddlers, using the PT MVPA cut-point of ≥420 counts⋅15 s^−1^ resulted in no significant difference in time spent in MVPA compared with direct observation [25]. Among 5–15 year-olds, a slightly higher cut-point of ≥573 counts⋅15 s^−1^ resulted in the best classification accuracy for MVPA [22]. The lower MVPA cut-point found in studies in younger children is plausible and might be due to physiological, biomechanical and structural factors, such as differences in gait parameters and body surface area to body mass ratios, which are thought to influence the association between accelerometer output and EE during childhood [26].

It is important to note that the results from this study are dependent on methodological decisions made in regards to defining SB and MVPA. Recently, there has been debate on the concept of SB and MVPA. SB has been defined as lying/sitting in some studies [4], [6], whereas other studies include lying/sitting and standing [7], [9], [27]. In addition, a consistent definition of MVPA is lacking. There has been a debate on the use of ≥3 versus ≥4 METs as the threshold for MVPA in children [28], [29], as well as differences in the use of EE units [4], [5] and direct observation systems [9], [30]. These methodological differences might explain why some studies reported higher SB and MVPA cut-points were more accurate compared to lower cut-points [27], [30]. To overcome this limitation in methodological studies it is important to reach agreement on the definitions of SB and MVPA in young children.

This study has several limitations. Due to the calorimeter sampling frequency and the time lag that exists when measuring EE in large volumes, it was not possible to measure EE in time blocks shorter than 10 min [13]. The room calorimeter is a confined space and the children followed a standardized activity protocol, limiting the ability to represent children’s free-living intermittent PA patterns. However, due to the small size and stature of the children, the limited space may have had less influence on their activity behavior than might be the case in older children or adults. In addition, as it was not feasible to ask preschool-aged children to fast overnight before completing a 2.5-hour activity protocol no measures of basal metabolic rate were available. Therefore, the Schofield equation [15] was used as a proxy measure of predicted basal metabolic rate which might have influenced the results. However, the Schofield equation [15] has been shown to be valid for estimating basal metabolic rate in preschoolers [31] and has been used for the same purpose in activity monitor validation studies in older children [22], [24], [32]. The proportion of data classified as valid when using EE combined with direct observation as criterion measure was low, especially for MVPA. This was due to the strict screening protocol used to reduce potential misclassification error from including, for example, data points in the MVPA category that may have been LPA (e.g. transitions between activities). However, our findings were essentially consistent with those from analyses where direct observation was used as the only criterion measure and very little data were excluded, supporting the overall conclusion.

This study had several strengths. The sample of 4–6 year old children was relatively large and evenly distributed by sex, and approximately representative with regards to weight status. Additionally, this accelerometer validation study is one of very few in young children that have used EE as criterion measure [4], [5], [24]. As EE was measured using a room calorimeter, children’s movements were not limited by wearing a facemask and the weight of a portable device. Wearing a facemask may not be tolerated by all young children, potentially impacting on how a given activity is performed. Conducting PA intensity classification analyses using only direct observation as a criterion measure as well as EE in combination with direct observation reduces the impact of the potential limitations associated with each of the methods. Last, the activity protocol used in this study complied with current best practice recommendations for activity monitor validation studies [33] as the protocol included a variety of child specific and developmentally appropriate ambulatory and non-ambulatory activities, ranging in intensity from SB to MVPA.

In summary, when measuring energy expenditure during MVPA, researchers may consider using the PT equation. However, neither the PT or PU equations, accurately predicted EE across all intensities, and therefore we do not recommend using these to predict EE in 4–6 year old children over a broad range of intensities. When assessing the prediction of PA intensity, EV resulted in good classification accuracy for SB, whereas the highest classification accuracy for MVPA was achieved when using PT. When classifying SB, LPA, and MVPA in 4–6 year old children, we recommend using ≤25counts⋅15 s^−1^, 25–419 counts⋅15 s^−1^, and ≥420 counts⋅15 s^−1^, respectively.
