# Correction: Predictive Validity and Classification Accuracy of ActiGraph Energy Expenditure Equations and Cut-Points in Young Children

**DOI:** 10.1371/annotation/c26dd8e7-d512-4255-a80c-f3bf56c1ac0d

**Published:** 2014-01-22

**Authors:** Xanne Janssen, Dylan P. Cliff, John J. Reilly, Trina Hinkley, Rachel A. Jones, Marijka Batterham, Ulf Ekelund, Søren Brage, Anthony D. Okely

In Figure 2, the asterisk (*) for statistically significant is missing on a number of bars. The correct version of Figure 2 can be found here: http://plosone.org/corrections/pone.0085130.g002.cn.tif

